# Compensatory Growth Impairs Adult Cognitive Performance

**DOI:** 10.1371/journal.pbio.0040251

**Published:** 2006-07-18

**Authors:** Michael O Fisher, Ruedi G Nager, Pat Monaghan

**Affiliations:** **1**Division of Environmental Evolutionary Biology, Institute of Biomedical and Life Sciences, University of Glasgow, Glasgow, United Kingdom; Cornell UniversityUnited States of America

## Abstract

Several studies have demonstrated that poor early nutrition, followed by growth compensation, can have negative consequences later in life. However, it remains unclear whether this is attributable to the nutritional deficit itself or a cost of compensatory growth. This distinction is important to our understanding both of the proximate and ultimate factors that shape growth trajectories and of how best to manage growth in our own and other species following low birth weight. We reared sibling pairs of zebra finches on different quality nutrition for the first 20 d of life only and examined their learning performance in adulthood. Final body size was not affected. However, the speed of learning a simple task in adulthood, which involved associating a screen colour with the presence of a food reward, was negatively related to the amount of growth compensation that had occurred. Learning speed was not related to the early diet itself or the amount of early growth depression. These results show that the level of compensatory growth that occurs following a period of poor nutrition is associated with long-term negative consequences for cognitive function and suggest that a growth-performance trade-off may determine optimal growth trajectories.

## Abstract 2

The speed with which adult zebra finches learned a new association task inversely correlates not with the degree of nutritional deprivation experienced early in life, but with the level of compensatory growth.

## Introduction

It has recently been recognised that many organisms are capable of adjusting their phenotypic development to mitigate at least some of the potentially negative effects of poor early nutrition [ 1, 2]. While it has also been recognised that these adjustments may themselves carry costs, possibly not evident until later in life, the processes responsible are poorly understood [ 3– 6].

A widespread example of such a mitigating response is “catch-up” or “compensatory” growth. Following an episode of poor nutrition during which growth is depressed, individuals can then accelerate or prolong their growth such that their external morphology at adulthood is indistinguishable from that of nonaffected individuals [ 4, 7, 8]. That many organisms have the capacity to grow faster than they generally do suggests that growth rate is optimised rather than maximised, varying in response to the balance of associated fitness costs and benefits [ 4, 5, 7, 9]. While offsetting disadvantages resulting from small adult size, compensatory growth might carry costs operational over varying time scales [ 10– 13] but nonetheless be favoured given the net fitness benefits relative to no compensation [ 4]. However, we still know very little about how such costs may occur, and hence about the selection pressures that shape optimal growth rates.

One important route through which early nutrition could profoundly influence subsequent performance is by effects on neural development and hence on cognitive ability [ 14]. Impairment of cognitive performance can obviously have pervasive effects on many important individual attributes, such as foraging ability, antipredator behaviour, mate acquisition, parenting, and the maintenance of physiological homeostasis through appropriate behavioural responses to changing environmental circumstances. Several studies in birds and mammals have provided evidence that poor nutrition in early life can adversely influence neural development, with, in the case of birds, adverse consequences for song learning [ 15– 19] and, in humans, subsequent intelligence quotients [ 20– 24]. However, the role played by the level of compensatory growth that occurs following the episode of poor nutrition has been little studied. In humans, it has recently been found that full-term babies born at low birth weight, and in whom subsequent compensatory growth was induced through provision of higher-quality feed (protein, mineral, and vitamin enriched), showed reduced cognitive performance at the end of the enrichment period (9 mo of age) relative to a control group not receiving such enrichment [ 25]. Moreover, the group most negatively affected was that which showed the greatest compensatory growth, in this case, girls [ 25, 26]. This suggests that the level of compensatory growth that occurs following nutritional deficit may be very important in determining aspects of early cognitive performance.

In examining the links between compensatory growth and cognitive abilities, it can be difficult to control confounding variables both during and after the manipulation period. Even within an experimental treatment, the changes in growth trajectories that occur may vary between subjects, and it is thus important to examine growth responses at the individual level and relate these to individual cognitive performance. Furthermore, it can be difficult to follow effects of growth on performance in the long term, especially in humans. Altricial birds offer a useful system in this context since they hatch in a relatively embryonic state; food is then provided by the parents without further processing, making it possible to directly manipulate the quality of nutrition available. The growth period is clearly defined and relatively short, and birds generally have good learning abilities and are widely used in learning studies.

Using same-sex sibling zebra finches Taeniopygia guttata exposed to different nutritional environments during the early posthatching period, we measured the degree of growth depression that occurred during an episode of nutritional deficit in early life and the degree of compensatory growth when normal nutrition was restored. We then tested whether subsequent learning performance in adulthood was linked to variation in these early growth trajectories.

## Results

### Early Nutrition and Growth

The zebra finch is a size-monomorphic species [ 27], and there were no sex differences in growth rates during either the deficit (day 0 to 20, *t*-test: *t*_32_ = 0.81, *p* = 0.42) or postdeficit periods (day 20 to 70, *t*_32_ = 1.29, *p* = 0.21). We therefore pooled the pairs of brothers ( *n* = 7) and pairs of sisters ( *n* = 10) for analysis. Figure 1 shows the average difference in the mass of the sib receiving a normal diet (N siblings) and that of its same-sex sib experiencing an early nutritional deficit (D siblings) before, during, and after the diet manipulation. Siblings allocated to the two early nutrition treatments did not differ in hatching mass (D birds = 1.0 ± 0.03 g; N birds = 1.0 ± 0.04 g; paired *t*-test: *t*_16_ = 1.16, *p* = 0.26). Nutritional deficit slowed the nestling growth of D birds relative to their N siblings so that, by the end of the diet manipulation period (20 d posthatching), offspring in the D group were on average significantly lighter (11.6 ± 0.40 g) than those in the N group (13.4 ± 0.26 g; paired *t*-test comparing siblings: *t*_16_ = 5.61, *p* < 0.001; Figure 1). In the postdeficit period (day 20 to 70 posthatching, all birds now on standard nutrition), most of the D birds then grew more than their N sibling; during this period D birds gained an additional 46.50 ± 4.78% of their day 20 body mass, compared to a 29.49 ± 2.91% average gain in the N birds (paired *t*_16_ = 3.07, *p* = 0.01). By day 70 posthatching, the birds were effectively fully grown. D birds (16.8 ± 0.46 g) were no longer significantly lighter than their N siblings (17.4 ± 0.52 g; paired *t*-test: *t*_16_ = 1.23, *p* = 0.24; Figure 1). Body mass changed very little thereafter; when the birds were weighed again following attainment of sexual maturity (around 100 d), body mass had changed on average 3% in the D birds and 5% in the N birds, and this change did not differ between them ( *p* = 0.28), and probably represents fat deposition rather than tissue growth.

**Figure 1:**
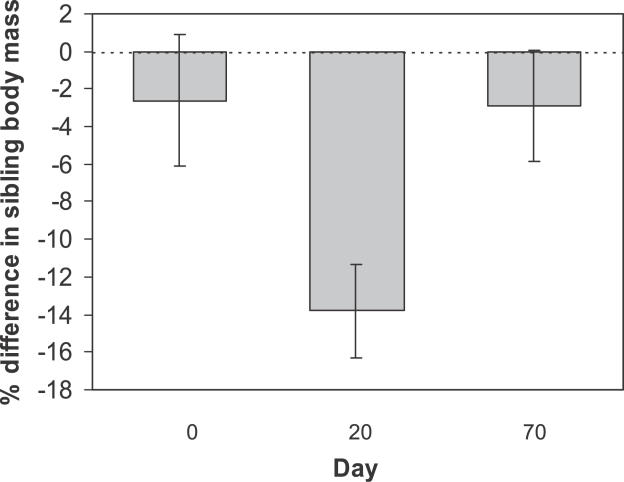
The Mean Difference in Body Mass between Pairs of Same-Sex Siblings at Different Stages of Development The differences were calculated as the body mass of the D sib, which experienced the early nutritional deficit minus that of the N sib reared under normal conditions, and are expressed as a percentage of the N sib's body mass. Thus negative values indicate that the D sib is lighter than its N sib.

However, there was substantial variation in the extent to which growth slowed in response to the dietary deficit regimen and in the degree of catch-up growth that occurred following resumption of normal feeding (coefficient of variation of within sibling pair differences in instantaneous growth rate between 0 and 20 d = 75.5% and between 20 and 70 d = 90.6%). Furthermore, the D birds that showed the strongest reductions in growth during the deficit period, relative to their N siblings, were not the same birds that produced the most extreme compensatory responses once the deficit period ended (correlation between sib differences in day 0 to 20 instantaneous growth rate and day 20 to 70 instantaneous growth rate: *r*_15_ = −0.31, *p* = 0.23). This allows us to separate the effects on cognitive abilities of the degree of slowing of growth during the period of nutritional deficit from effects associated with the degree of later compensatory growth.

### Early Nutrition and Adult Learning Ability

All birds reduced the number of errors they made over the course of the 16 associative learning trials, and thus all birds seemed capable of eventually learning the task; this was not influenced by biological or foster parent or early diet ( Figure 2). To examine differences in the speed of learning of siblings in relation to growth pattern, we used the sibling difference in the number of trials it took them to achieve two trials without error and sibling differences in growth. The difference in learning speed between siblings was strongly related to the extent to which growth compensation occurred and not related to the magnitude of growth depression during the deficit period (multiple regression: sibling differences in growth from day 20 to 70: *F*_1,14_ = 8.03, *p* = 0.01; sibling differences in growth from day 0 to 20: *F*_1,14_ = 0.02, *p* = 0.90, Figure 3A and 3B). The D birds that showed a strong compensatory growth response relative to their N sibling performed relatively poorly on the associative learning task ( Figure 3B). Learning speed was not influenced by early diet itself (paired *t*_16_ = 1.03, *p* = 0.32) or by body mass at testing ( *r*_32_ = 0.26, *p =* 0.14). If the sib groups are considered separately, then in neither group is there any significant relationship between learning speed and growth rate during the deficit period (N sibs *p* = 0.17, D sibs *p* = 0.68); only in the D group was there a relationship between learning speed and growth rate during the compensatory period (N sibs r _15_ = 0.02, *p* = 0.95, D sibs r _15_ = 0.62, *p* = 0.008). Thus, the data clearly demonstrate that those birds that grew relatively fast when normal nutrition was restored following a period of early deficit had poor learning performance in adulthood.

**Figure 2:**
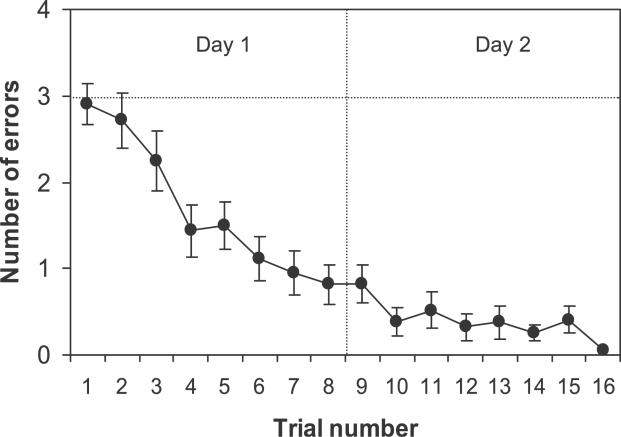
The Mean (± SE) Number of Errors per Trial Made on the Associative Learning Task by Experimental Birds (see Materials and Methods) All birds (i.e., both sexes and birds from both early nutrition treatment groups) are included ( *n* = 34). The dotted line indicates the number of errors expected by chance. The dashed line denotes a 24-h retention interval (i.e., trial 9 was the first trial of day 2). Sex, early diet, and family of origin did not influence the pattern of decline in errors with trial number (mixed model using a binomial error distribution where trial number was a repeated measure within bird identity [random effect]: trial number: *z* = 14.07, *p* < 0.0001; bird identity: *z* = 0.13, *p* = 0.45; family of origin [random factor]: *z* = 1.12, *p* = 0.13; sex: *F*_1,13.4_ = 0.05, *p* = 0.82; early diet: *F*_1,13.7_ = 0.74, *p* = 0.40), nor did the foster family in which the experimental birds were reared affect learning performance ( *F*_18, 15_ = 0.56, *p* = 0.88).

**Figure 3:**
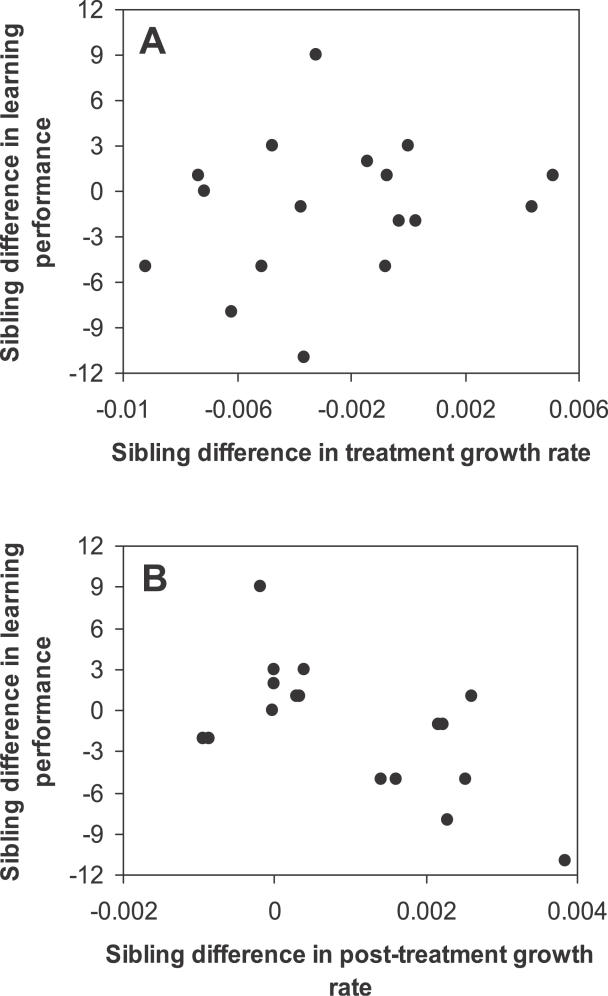
Relationship between Compensatory Growth following a Period of Low-Quality Early Nutrition and Associative Learning Performance in Adulthood Within-sibling differences (D sib – N sib) in the growth increment from (A) day 0 to 20 and (B) day 20 to 70 and learning performance are plotted so that each data point represents a sibling pair. Positive values for growth indicate that the D sib grew faster during the postdeficit period than its same-sex N sib that had experienced normal nutrition throughout growth. The learning performance was scored on the basis of the number of trials it took them to achieve two trials without error. Birds that did not reach this criterion obtained a score of zero and the fastest learners obtained the highest score. The figure shows the difference in score between the sibling pairs, and negative values mean that the D sib had a poorer learning performance than its N sib. There was no correlation between the intersib difference in growth during the deficit period (0 to 20 d) and learning performance in adulthood (A: *r*_15_ = 0.10, *p* = 0.70); however, the faster the D sib grew relative to its N sib when normal nutrition was restored, the poorer was its learning performance in adulthood (B: *r*_15_ = 0.63, *p* = 0.007). In this relationship, no point had a large influence (Cook's distance for all points <0.4).

## Discussion

Episodes of poor early nutrition have been linked to impairment of performance in adulthood in a number of taxa [ 6, 28– 31]. The extent to which such impairments are associated with a shortage of nutrients and consequent slowing of growth during crucial stages of development, or a negative consequence of the compensatory growth that often follows the period of poor nutrition, is unclear. It is generally very difficult to separate the two, since the nutritional deficit induces compensatory growth if normal nutrition is restored and thus the two are closely linked. However, distinguishing between the two is important if we are to understand what determines optimal growth rate. Individuals do vary in their responses to particular developmental conditions, such that, even within a particular experimental treatment, both the magnitude of growth depression and growth compensation can vary amongst individuals, possibly due to genetic or other developmental differences [ 4, 5]. In our experiment, we manipulated early posthatching nutrition in zebra finches, controlling for genetic effects by using siblings, and monitored the growth responses of individuals both during the deficit and after the restoration of normal levels of nutrition. Those individuals in the deficit group that showed the greatest growth depression relative to their genetic sibling were not those that showed the most growth compensation. This allowed us to separate to some extent the magnitude of the early deficit from the degree of compensatory growth that then occurred. Furthermore, we also standardised the environment and level of nutrition the birds experienced until testing at adulthood. Our data on zebra finches show that it is the magnitude of growth compensation in response to the nutritional deficit, rather than the early diet itself or the degree of growth depression during the deficit, that is related to subsequent learning performance in adulthood.

Another possibility is that our results are a consequence of some underlying relationship between activity levels and cognitive performance, in that if the slower learners are generally less active than brighter birds, the former may put on more mass than the latter. In our experimental conditions, there is in fact rather little scope for substantial differences in activity levels amongst the young birds, since they are bred in nest boxes in cages and fed by their parents. However, more important, our data suggest that this explanation is unlikely. If there was a general relationship between activity levels (and thereby mass gain) and cognitive ability, we would then expect to see this relationship throughout the growth period, i.e., also during days 0 to 20. However, during this period there was no relationship between growth rate and adult learning performance when examined in terms of sib differences, or in the D or in the N birds when these are considered separately. We would also expect to see this general relationship within N as well as the D birds in the later growth period, days 20 to 70. This is not so—the relationship between growth and learning performance only occurs in those birds that experienced an early nutritional deficit and were then undergoing compensatory growth to differing degrees.

What we have found is that the more compensatory growth following an early deficit, the poorer is the learning performance in adulthood. This is in accordance with effects reported over a relatively short-term study of human infants involving similar relatively small differences in the quality of the food provided (1.45% protein formula milk feed compared with feed containing 1.85% protein plus enriched vitamin and mineral content) [ 25]. In Morley et al.'s human study, the difference in cognitive performance recorded in babies at 9 mo (the end of the early nutrition treatment period) was not detected at 18 mo. In our study, effects of compensatory growth on cognitive performance, measured as the speed of learning of a relatively simple task, were detectable in adulthood. The effects in human offspring may be more transient, be more difficult to measure at later stages, or have been offset by environmental differences. It is possible, for example, that mothers with children whose cognitive development seems slow may provide more stimulation that has a beneficial effect. It is obviously difficult to control this experimentally.

While it has been suggested that development of the central nervous system is prioritised during development [ 32], it is not entirely buffered from nutritional effects. Mammalian experimental studies have shown that nutritional conditions during growth and development can have long-term effects on brain structure and function [ 15, 21]. In birds, early nutrition has been found to affect the development of song centres in the male brain [ 18, 19]. However, even though considerable neurogenesis is known to occur in non-song brain structures during juvenile and adult life [ 33], no previous studies have experimentally examined the impact of early growth on general cognitive ability in birds. This study suggests that compensatory growth can have long-lasting negative effects on cognitive performance. There are a number of ways in which such effects could come about. In this study, it was the speed of learning in particular that was affected, and all birds did eventually learn the task. This slow learning could involve behavioural, endocrinological, and neural changes. A diversion of resources from neural development to fuel growth of other body parts may occur and/or there may be a shift in investment in tissue growth at the expense of maturation as a consequence of a growth/maturation trade-off [ 34, 35]. Prolonged stress during a period of compensatory growth may produce chronically elevated levels of corticosterone, which could influence behaviour; it is known to result in irreversible damage to the hippocampus, a brain region closely associated with learning and memory [ 36]. Furthermore, chronically high levels of corticosterone could potentially have more widespread effects in the avian than the mammalian brain, given the substantial neurogenesis that can occur in adulthood in birds [ 37]. The vertebrate brain also contains a large number of polyunsaturated fatty acids that are especially susceptible to free radical damage [ 38], which may increase during rapid growth. Any such negative effects of growth pattern on cognitive performance are likely to have far reaching consequences for individual fitness. Further work is required in order to identify the cause of the slower learning speed, both at the behavioural level, since there are many different ways in which the slower association between screen colour and food reward could come about, and at the level of brain growth and function. Understanding how growth rate may influence organism function is an important area of research in both the context of understanding the evolution of growth rates and developing appropriate procedures for managing growth following low birth weight.

## Materials and Methods

### Early nutritional deficit.

In order to evaluate the effects of early growth, while allowing for genetic effects, we compared the cognitive performance of genetic full siblings of the same sex reared under different qualities of posthatching nutrition. Zebra finches with previous breeding experience were randomly paired and allowed to nest in individual breeding cages (60 cm × 45 cm × 40 cm). All birds were provided with the standard diet recommended for captive finches of ad libitum seed (J.E. Haith Ltd, Cleethorpes, United Kkingdom; foreign finch mixture, containing 11.6% protein); a vitamin and mineral supplement (Daily Essentials 2; The Birdcare Company, Nailsworth, United Kingdom), plus Haith's conditioning food (13.6% protein), was provided three times a week. Zebra finch parents carry food provided in the cage to the nestlings in the nest box for about the first 20 d posthatching, following which the chicks gradually begin to fledge, leaving the box and taking food in the cage themselves. It is thus not possible to control or measure directly the food fed to each chick. We therefore manipulated the quality of the food available to the chick-rearing parents. When the first chick in each nest hatched, nests were allocated randomly to the two early nutrition treatment groups, one of which involved provision of the high-quality rearing diet normally provided during chick rearing and the other, a lower-quality diet. Nests in the normal diet (N) group were provided with the ad libitum mixed seed, and the vitamin and conditioning food was provided daily, together with a homogenised egg mixture containing around 12% protein. Nests in the low-quality early diet treatment were provided only with ad libitum mixed seed and water (Early Deficit Group-D), and none of the higher-protein conditioning food or the vitamin and mineral supplement. These diets were maintained for the first 20 d posthatching. From then on, all nests were provided with the standard diet as described above for parents prior to chicks hatching. All birds were then maintained on this standard diet for the remainder of the experiment.

Since we did not know the sex of chicks at hatching, we ensured that pairs of same-sex siblings experienced different dietary regimens by using the following experimental protocol. On the day of hatching, each genetic sibling was fostered to a different nest, with sibs being fostered alternately to D nests and N nests. Fostering was arranged so that, after chicks had been moved, each foster nest contained three unrelated chicks. If it was later found that more than one same-sex sibling had been allocated to the same treatment ( *n* = 4 cases), one of these birds was chosen at random for use in the learning study. This rearing procedure therefore also controlled for any effects of fostering, since no chicks were reared by their genetic parents. Each chick was weighed on the day it hatched and again at 20 and 70 d posthatching; instantaneous growth rates (log mass _2_ – log mass _1_/t _2_ – t _1_) for the period of nutritional deficit (day 0 to 20) and the posttreatment period (day 20 to 70) were calculated. From 35 d posthatching onward, offspring can be sexed from plumage [ 27], and they were then removed from their foster nest and placed in single-sex groups of four to six young birds, with same-sex siblings being in the same group. Seventeen pairs of same-sex siblings that had experienced different early nutrition were available for study in adulthood (ten sister-sister pairs, seven brother-brother pairs).

### Learning task.

All 34 birds were given an associative learning task in adulthood (approximately 150 d old at testing). These trials were conducted in a circular foraging arena (height 40 cm, diameter 150 cm) under full spectrum lighting (bird lamp' Arcadia, Croydon, United Kingdom). The arena contained seven corridors leading from a central area. At the end of each corridor was a white screen behind which cups containing seed were placed. Initially, birds were given shaping sessions to ensure that they were familiar with the arena and the food cups. Once all birds were readily feeding from the cups, birds were then entered into trials in which they were required to learn an association between the colour of a screen and the presence of food. In this situation, only one of the screens, coloured yellow, now had food behind it; the other six screens were black. The location of the yellow screen was randomly altered between trials. Each bird was given eight consecutive trials per day, for two consecutive days, before the first feeding in the morning. Each trial consisted of a 10-min acclimation period (inside a pulley-operated mesh holding chamber in the centre of the arena) to enable the bird to recover from handling; this was followed by a search phase in the arena. During the search phase, the number of errors (i.e., the number of unrewarded screens visited) that a bird made prior to finding the rewarded tray was recorded. Once the bird located the rewarded tray, it was allowed to feed for 30 s and then removed from the arena. Following each feeding from behind the yellow screen, the bird was given a 10-min rest interval prior to the start of the next trial. The amount of feeding was such that birds remained hungry throughout the consecutive trials on each day, following which they were returned to their home cage and allowed to feed normally.
